# The utility of dual energy computed tomography in the management of axial gout: case reports and literature review

**DOI:** 10.1186/s41927-020-00119-6

**Published:** 2020-05-08

**Authors:** Jeremy X. Wang, Beverly Ng, Haesung Bak, David Spencer, Nicholas Manolios, Peter K. K. Wong

**Affiliations:** 1 Department of Rheumatology, Westmead Hospital, Westmead, Sydney, NSW 2145 Australia; 2 Sydney Medical School, University of Sydney, Sydney, NSW Australia; 3 Rural Clinical School, Coffs Harbour, Faculty of Medicine, University of New South Wales, Sydney, Australia

**Keywords:** Spinal gout, Back pain, Dual energy computed tomography, Diagnosis

## Abstract

**Background:**

Severe spinal pain is an unusual presentation of gout. Due to its rarity and the difficulty of obtaining joint fluid or tissue for crystal analysis, dual energy computed tomography (DECT) may be a useful imaging modality in the management of axial gout.

**Case presentation:**

Two patients independently presented to a major teaching hospital with severe spinal pain subsequently shown to be due to gout. The first patient presented with back pain and fevers and was initially thought to have lumbar facet joint septic arthritis. The second case presented with severe back pain. In both cases, DECT suggested monosodium urate deposition in spinal tissues as the cause of their presentation.

**Conclusions:**

Axial gout should be considered in the differential diagnosis of severe spinal pain. A DECT study may be a useful diagnostic tool in the management of spinal gout.

## Background

Gout is caused by monosodium urate (MSU) crystal deposition in synovial joints and is the most common acute inflammatory arthritis [1]. Most gouty attacks present with an acute peripheral inflammatory arthritis - axial involvement is less common [2]. The diagnosis of spinal gout can be challenging due to the anatomical location, making access to a diagnostic histologic sample difficult. Dual energy computed tomography (DECT) utilises x-rays at two energy levels and provides additional information compared to a conventional CT scan regarding tissue composition, artefact reduction and image optimisation. When used as a non-invasive modality to demonstrate MSU crystal deposition, the reported sensitivity and specificity is 78–100% and 89–100%, respectively [3–7]. We present two cases of gout involving the axial skeleton in which DECT significantly assisted management.

## Case presentation

### Case 1

**Fig. 1:**
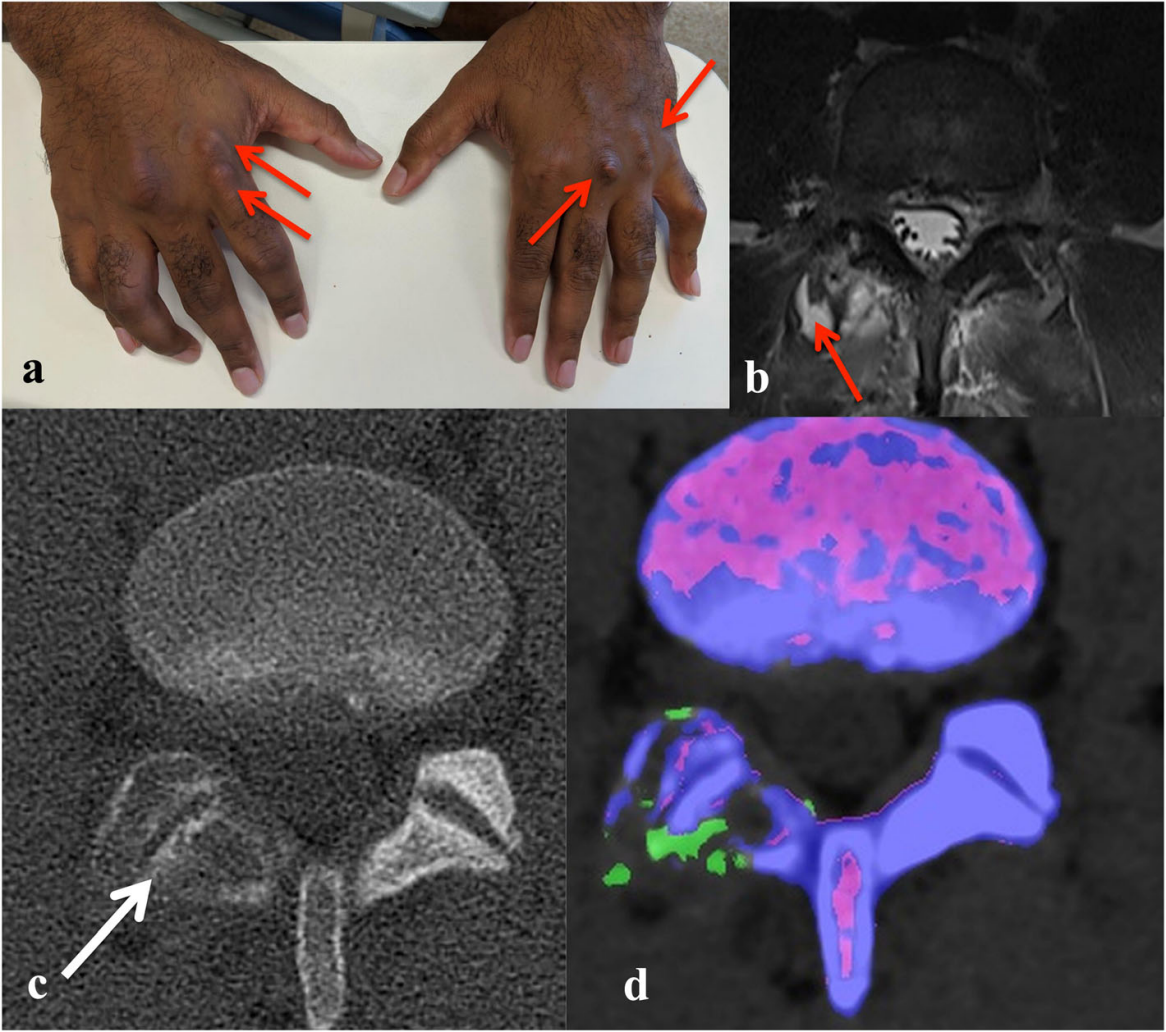
**a** A 32-yo male Pacific Islander with probable gouty tophi affecting both hands (red arrows). **b** Lumbar spine MRI (T2, axial view) image reported as showing an abscess surrounding the right L4/5 facet joint (red arrow). Axial CT image of lumbar spine showing **c** erosions (white arrow); and **d** corresponding DECT image (Siemens Somatom Force™) showing monosodium uric acid crystal deposition (green)

A 32-year old male Pacific Islander presented to the Emergency Department with recent onset acute severe lower back pain and new onset left knee pain associated with fevers and chills. There were no bowel or bladder symptoms. Past medical history included tophaceous gout and asthma. Current medications included allopurinol 300 mg daily with variable adherence, and a salbutamol inhaler. He worked as a security officer, did not smoke and occasionally consumed alcohol. On examination, he was febrile at 38.5 °C with multiple probable tophi on the dorsum of both hands (Fig. 1a). The left knee was tender and warm with a moderate effusion. Neurological examination was unremarkable.

Initial investigations showed a peripheral neutrophilia of 21 × 10^9^/L (normal range: 3.7–9.5 × 10^9^/L) and an elevated C-reactive protein (CRP) of 250 mg/L (normal range < 5 mg/L). Serum creatinine and uric acid level were 122 μmol/L (normal range 60-110 μmol/L) and 0.53 mmol/L (normal range 0.16–0.41 mmol/L), respectively. Synovial fluid aspiration of the left knee was performed and 15mls of inflammatory-appearing synovial fluid was sent for analysis. This revealed a white cell count of 33 × 10^9^/L (100% polymorphs). Monosodium urate crystals were identified in the knee fluid and no organisms were seen on Gram stain. Subsequent culture of the knee synovial fluid and blood was negative. Magnetic resonance imaging (MRI) of the spine to investigate the severe back pain was reported as showing right L4/5 facet joint septic arthritis with abscess formation (Fig. 1b). Given the MRI report, he was empirically started on intravenous cephazolin 2 g every 8 h. A CT-guided aspiration of the right L4/5 facet joint collection was performed. No organisms were seen on Gram stain of the fluid obtained. Subsequent cultures were negative, but MSU crystals were seen.

A DECT was performed to assess the extent of MSU crystal deposition in the spine (Fig. 1c, d). This showed multiple well-defined focal erosions at the right L3/4, L4/5 and L5/S1 facet joints, with dual energy attenuation characteristic of MSU deposition (Fig. 1c and d). As cultures of the knee and spinal collection fluid remained negative at 48 h, antibiotics were stopped. Initiation of prednisone 25 mg daily and colchicine 0.5 mg twice daily resulted in marked improvement in back pain. Non-steroidal anti-inflammatory drugs (NSAIDs) were not used due to the renal impairment. Allopurinol was re-initiated while he was on prednisone and up-titrated with a target serum urate level of < 0.30 mmol/L.

### Case 2

A 74-year old Caucasian male was admitted for investigation of acute on chronic renal failure on a background of type 2 diabetes, hypertension and ischaemic heart disease. Regular medications included carvedilol, telmisartan, hydralazine, furosemide, spironolactone, ivabradine, sitagliptin and metformin, with meloxicam as required. He denied recent heavy alcohol intake. On closer questioning, he reported a six-week history of lower back pain and long-standing bilateral knee pain. There was no previous history of gout. On examination, multiple lesions consistent with gouty tophi were present over the extensor aspect of both elbows and left Achilles tendon. Bilateral knee effusions with low-grade synovitis were present.

**Fig. 2:**
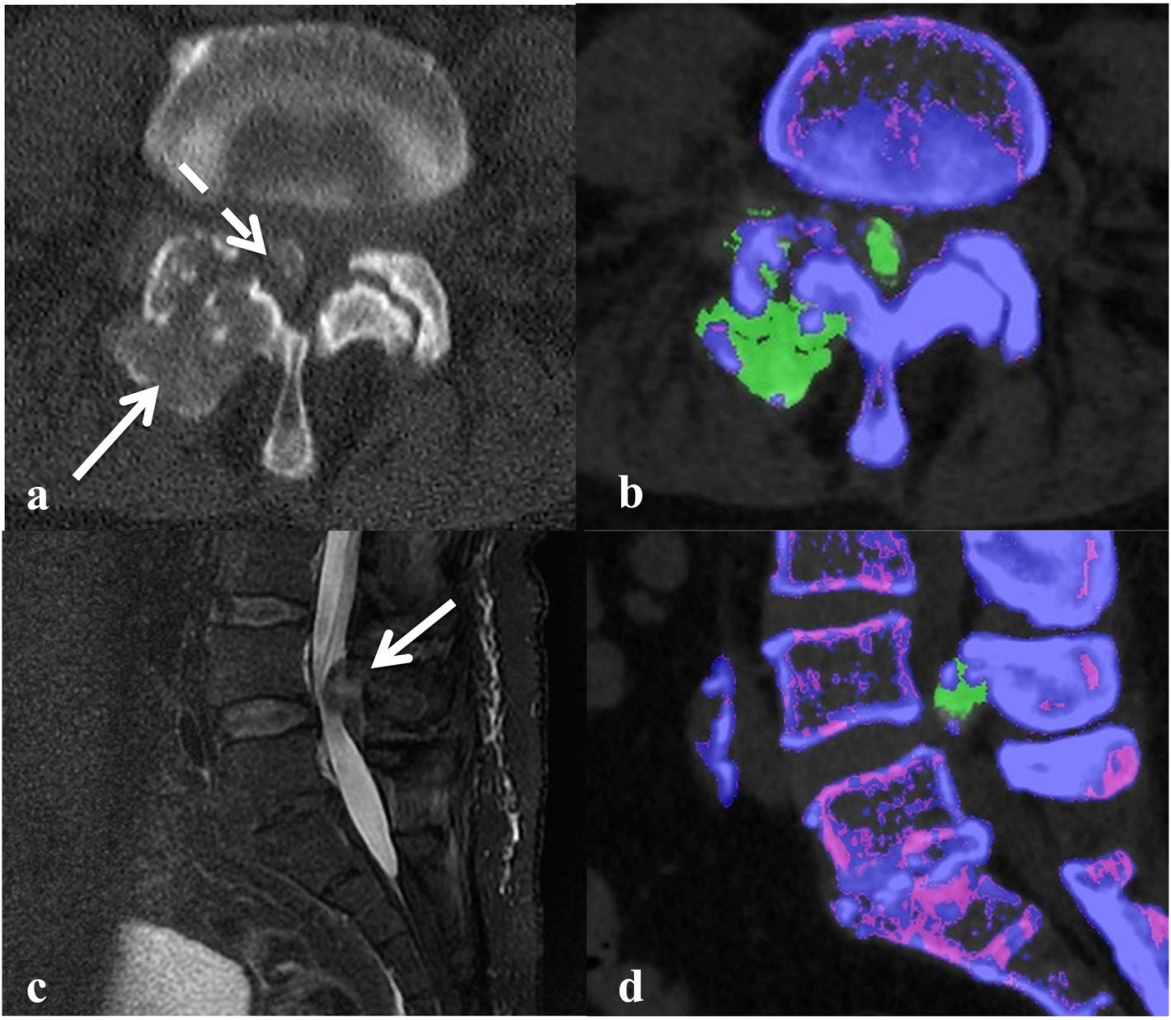
**a** Axial CT image showing right L4/5 facet joint erosion (white arrow) with calcified peri-articular mass encroaching on lumbar canal (dashed white arrow), and **b** corresponding DECT image (Siemens Somatom Force™) showing monosodium uric acid crystal deposition (green). **c** Sagittal T2–fat suppressed MRI image (General Electric 1.5 T Signa Excite™) of the lumbar spine showing the soft tissue mass seen in **a** and **b** causing marked lumbar canal stenosis, and **d** corresponding DECT image (Siemens Somatom Force**™**) showing attenuation consistent with monosodium uric acid crystal deposition

Initial investigations were as follows: serum creatinine 406 μmol/L (usual baseline 104 μmol/L, normal range 60-110 μmol/L), serum uric acid 0.71 mmol/L (normal range 0.16–0.41 mmol/L) and CRP 140 mg/L (normal range < 5 mg/L). A CT and MRI scan of the lumbar spine showed an erosive arthropathy involving the right L4/L5 facet joint associated with a partially calcified peri-articular mass causing marked spinal canal stenosis and compression of the descending right L5 nerve root (Fig. 2a and c).

Given the history of chronic renal disease and presence of peripheral tophi, DECT was performed which demonstrated attenuation of the soft tissue mass at the right L4/5 facet joint consistent with MSU deposition (Fig. 2b and d). A diagnosis of axial gout was made. The patient was commenced on prednisone 25 mg daily for the management of acute gout. Non-steroidal anti-inflammatory drugs and colchicine were not used due to the severe renal impairment. Urate-lowering therapy with allopurinol was initiated, and febuxostat added as it was thought the target serum urate level was unable to be reached on allopurinol monotherapy. There was marked rapid symptomatic improvement. On discharge, the serum urate level was 0.35 mmol/L (normal range 0.16–0.41 mmol/L).

## Discussion and conclusion

Axial pain is an unusual presentation of gout. Whilst asymptomatic MSU deposition in the axial skeleton can occur in healthy subjects [8], it can also result in severe back pain [2, 9], as seen in both our cases. Most patients with axial gout have a history of tophaceous gout affecting the peripheral joints and/or hyperuricemia [9]. Both our patients had severe tophaceous gout at presentation. However, Case 2 was previously undiagnosed, possibly due to poor health literacy and lack of attendance at his general practitioner. In Case 1, poor medication adherence was responsible for accumulation of gouty tophi and the presenting severe flare.

In most published cases of spinal gout, the diagnosis was confirmed on synovial fluid analysis obtained by surgical or radiological aspiration of the lesion [2]. However, the anatomic location and limited availability of spinal surgical or interventional radiology expertise may make this challenging. Dual energy CT is a recently developed technology, which first saw clinical utility in 2005 [10]. It has better tissue characterisation and artefact reduction compared to conventional CT [10]. The reported sensitivity and specificity of DECT for detection of MSU deposition is 78–100% and 89–100%, respectively [3–7]. Despite the relatively widespread use of DECT for detection of MSU crystals in peripheral joints, there have only been a handful of case reports of its use in spinal gout [2, 9, 11–15]. Due to the high-quality images and ability to assess soft tissue structures, MRI is often the initial imaging modality used to investigate back pain, especially in the presence of neurologic impairment. However, MRI is unable to specifically identify MSU deposition. In fact, in Case 1, the initial MRI findings suggested an incorrect diagnosis of septic arthritis. In the right clinical context, DECT could potentially avoid an invasive diagnostic aspiration/biopsy, especially in those patients who may be medically unfit due to co-morbidities. For example, Case 2 did not undergo a diagnostic spinal aspirate and was successfully managed with medical therapy, as there were no clinical features of infection. However, in Case 1, the history of high fevers and chills necessitated exclusion of spinal infection by a CT-guided aspirate of the soft tissue mass (Fig. 1b).

Gout should be considered in the differential diagnosis of severe back pain – especially in the presence of a previous gouty attack and/or tophi. These two cases demonstrate the ability of DECT to detect MSU crystal deposition in the spine. Depending on the clinical setting, this can be a useful non-invasive diagnostic tool.

## Data Availability

Not applicable.

## Abbreviations

CRP: C-reactive protein
CT: Computed tomography
DECT: Dual energy computed tomography
MRI: Magnetic Resonance Imaging
MSU: Monosodium urate
NSAID: Non-steroidal anti-inflammatory drug
